# Synergistic impact of plasma albumin and cognitive function on all-cause mortality in Chinese older adults: a prospective cohort study

**DOI:** 10.3389/fnut.2024.1410196

**Published:** 2024-07-24

**Authors:** Zhi-qiang Li, Xin-xin Liu, Xue-feng Wang, Chen Shen, Feng Cao, Xin-miao Guan, Ying Zhang, Jian-ping Liu

**Affiliations:** ^1^Centre for Evidence-based Chinese Medicine, Beijing University of Chinese Medicine, Beijing, China; ^2^The National Research Center in Complementary and Alternative Medicine (NAFKAM), Department of Community Medicine, Faculty of Health Science, UiT The Arctic University of Norway, Tromsø, Norway

**Keywords:** plasma albumin, cognitive function, mortality, CLHLS, older adults

## Abstract

**Background:**

Hypoalbuminemia and cognitive impairment (CI) each independently increase the mortality risk in older adults. However, these two geriatric syndromes can occur simultaneously. In community-dwelling older adults, is the combination of hypoalbuminemia and CI linked to a higher mortality risk than either condition alone?

**Objective:**

We aimed to investigate the association between plasma albumin, cognitive function, and their synergistic effect on mortality in Chinese community-dwelling older adults.

**Methods:**

Data from the Chinese Longitudinal Healthy Longevity Survey (2012) included 1,858 participants aged ≥65. Baseline assessments comprised albumin levels and cognitive status. All-cause mortality was confirmed through 2014–2018 surveys. Cox proportional hazards models assessed associations, and restricted cubic splines explored albumin-mortality relationship.

**Results:**

During a median follow-up of 48.85 months, 921 deaths. Albumin≥35 g/L *vs* < 35g/L [HR: 1.33 (95%CI, 1.10, 1.62)] and CI *vs* normal cognition [HR: 1.69 (95%CI, 1.43, 1.99)] independently predicted mortality. A dose–response relationship with mortality was observed for albumin quartiles (*p* < 0.001). Each SD increase in MMSE or albumin correlated with 22% and 15% lower mortality risk, respectively. Combined hypoproteinemia and CI increased the mortality risk by 155%, with a notably higher risk in males, those aged <85 years, and individuals living in rural areas. Interaction effects of albumin and CI on mortality were observed (*p* < 0.001). In the single CI group, older adults had a 61% increased risk of mortality in the hypoproteinaemia group compared with the albumin-normal group. Restricted cubic spline revealed a reverse J-shaped association, particularly for participants without CI. For individuals with CI, albumin levels were inversely associated with mortality risk.

**Conclusion:**

Hypoproteinemia and CI, individually and combined, increased all-cause mortality risk in Chinese older adults, with stronger effects observed in males, younger older adults, and those living in rural areas. These findings emphasize the importance of targeted adjustments and early nutrition programs in health prevention and clinical care for older adults.

## Introduction

As the proportion of individuals aged 65 and above in China continues to rise, constituting 14.2% of the population in 2021 according to the statistical yearbook issued by the Chinese government (1), the issue of cognitive decline emerges as a significant health concern. In a preliminary meta-analysis of 35 studies, the pooled prevalence of mild cognitive impairment (MCI) was 17.3% (95%CI: 13.8–20.8%) in community-dwelling older adults aged 60 years and older (2, 3), with the incidence varying due to factors such as mean age, education level, diagnostic criteria, cognitive tests used, and operationalization of these criteria. The prevalence is notably high in China (4). Cognitive impairment (CI) refers to various degrees of cognitive function damage caused by various reasons, ranging from MCI to dementia. CI not only adversely affects the ability of older individuals to live independently and their quality of life, elevating hospitalization rates and the burden on social care, but also contributes to mortality risk (5, 6). Cognitive function is influenced by multiple factors, and in recent years, an increasing number of researchers have spotlighted the connection between albumin levels and CI (7, 8).

Plasma albumin, the most abundant protein in human plasma, is a vital component produced by the liver. It plays a pivotal role in maintaining body osmotic pressure, enhancing immune function, and maintaining acid–base balance (9). In the progression of diseases, a relatively low level of albumin is closely associated with adverse outcomes, including ischemic heart disease, myocardial infarction, and ischemic stroke (10, 11). Independent studies on community-dwelling older adults in China and the Amsterdam longitudinal aging study independently have identified low serum albumin levels as an independent factor associated with cognitive performance (12, 13). The decline in albumin is not only a pathogenic marker but also closely related to mortality. Previous studies have established hypoalbuminemia as a high-risk factor for death, primarily focusing on specific diseases such as myocardial infarction, acute kidney injury, chronic kidney disease, and acute heart failure, as well as in hospitalized patients (14–18). However, there is limited exploration of the dose–response relationship between albumin levels and all-cause mortality, especially in the elderly. Therefore, further evaluation is essential to determine the predictive value of decreased albumin concentration for the risk of death in older adults.

Extensive research, both national and international studies, has investigated the distinct contributions of plasma albumin and CI, revealing their significant impact on the health of older adults and their heightened association with mortality in aging populations (5–8, 14–18). Hypoproteinemia commonly coexists with CI, and both conditions exhibit a proportional increase with advancing age (13, 19). Nevertheless, the combined impact of hypoalbuminemia and CI on mortality remains an under-explored domain, particularly among Chinese older adults. Additionally, the influence of cognitive status on the association between plasma albumin levels and mortality remains unclear. And there is no evidence on the combined effect of these conditions on mortality in community-dwelling older adults. Therefore, this study, utilizing data from the Chinese Longitudinal Healthy Longevity Survey (CLHLS), explores an intriguing and underexplored aspect of geriatric health–the combined impact of plasma albumin levels and cognitive function on all-cause mortality in Chinese community-dwelling older adults, along with potential underlying interactions. The research aims to unravel the intricate interactions between these factors and contribute valuable insights for the prevention and treatment of CI, considering the synergistic effects of different risk factors prevalent in aging populations.

## Materials and methods

### Study population

This study utilized data from the Healthy Aging and Biomarkers Cohort Study (HABCS), a biomarker sub-cohort derived from the CLHLS in 2012. The baseline participants were sourced from the eight longevity regions during the 6th wave (2012) of the CLHLS, with subsequent follow-ups conducted in the 7th (2014) and the 8th (2018) waves. These regions, identified by the Chinese Society of Gerontology in 2011 (20), are representative of one-third of the longevity areas, characterized by higher concentrations of the oldest-old, particularly centenarians (>7/100,000), and higher life expectancies. The comprehensive details and descriptions of the CLHLS have been exhaustively documented in previous literature (21, 22).

During the baseline survey, a total of 2,439 participants were involved in the 6th wave of CLHLS. Excluding 122 subjects due to missing data on plasma albumin and other biochemical parameters, we further excluded individuals younger than 65 years old (*n* = 84) and those without cognition data (*n* = 4). Additionally, 331 participants who lost to follow-up were excluded. Consequently, the final analysis included 1,858 older adults (aged≥65) with available biomarker and cognition data. This cohort in the final analysis comprised 457 octogenarians, 368 nonagenarians, and 438 centenarians. The flow chart of the study is presented in Supplementary Figure S1. Ethical approval was obtained from the Research Ethics Committee of both Duke University and Peking University (IRB00001052-13074). Written informed consent was obtained from all participants or their legal representatives.

### Cognitive function

Cognitive function was assessed using the Chinese version of the Mini-Mental Status Examination (CMMSE), a well-established, sensitive, and reliable scale widely employed for cognitive impairment screening (21–23). The Chinese adaptation of this instrument has undergone rigorous validation in research settings within the elderly community. The MMSE is a 30-point assessment tool that measures various cognitive functions, with higher scores indicative of better cognitive performance. Taking into account the level of education (24, 25), CI was defined as follows: participants with no formal education (MMSE score < 18); those with 1–6 years of education (MMSE score < 21); or individuals with over 6 years of education (MMSE score < 25).

### Plasma albumin

Trained medical personnel collected overnight fasting venous blood samples from all elderly participants. Within one hour after blood collection, plasma and blood cells were separated by centrifugation and then stored at-20°C. The samples were transported via cold chain to the Central Clinical Laboratory at Capital Medical University in Beijing. In addition, those blood samples were stored at −80°C until analyzed centrally. Procedures for the collection and shipment of blood samples were also described elsewhere (20). According to the plasma albumin level (26), we used both quartiles and a binary variable of particular significance value (normal plasma albumin≥35 g/L; hypoalbuminemia <35 g/L) for individual association analyses and applied the binary variable in the combined association analyses.

Blood plasma analyses, including creatinine, lipid profile [total cholesterol (TC), high-density lipoprotein cholesterol (HDL-C), low-density lipoprotein cholesterol (LDL-C)], triglyceride (TG), fasting blood glucose (FBG), high sensitivity c-reactive protein (hs-CRP), and serum uric acid (SUA) were analyzed by an automatic biochemistry analyzer (Hitachi 7,180, Swiss Roche Company, Tokyo, Japan) using commercially available diagnostic kits (Roche Diagnostic, Mannheim, Germany), and serum creatinine was determined by the picric acid method, BUN was determined by urease ultraviolet rate method, and blood uric acid was determined by uricase colorimetric method.

### Covariate

Covariate information was obtained through home interviews and baseline biochemistry tests (24, 25). Selected covariates included demographic factors such as age, gender (male/female), ethnicity (Han/others), residence (urban/rural), marital status (married and living with spouse/others), and education (0/1–6/>6). Lifestyle characteristics encompassed current smoking (yes/no), current alcohol drinking (yes/no), and current exercise (yes/no). Physical measurement indicators included BMI, central obesity, and ADL disability. Chronic conditions were assessed through self-reported and/or hospital-diagnosed hypertension, diabetes mellitus (DM), heart disease, cerebrovascular disease, respiratory disease, and arthritis. Fasting blood specimens were analyzed, including TG, TC, HDL-C, LDL-C, FBG, SUA, and hs-CRP.

BMI, calculated by dividing weight in kilograms by the square of body height, is expressed as kg/m^2^. Individuals were classified: non-overweight and obese (BMI < 24 kg/m^2^), overweight and obese (BMI ≥ 24 kg/m^2^). Central obesity was defined as waist circumference (WC) ≥ 85 cm in males and WC ≥ 80 cm in females. Hypertension was determined as systolic blood pressure (SBP) ≥ 140 and/or diastolic blood pressure (DBP) ≥ 90 mmHg and/or a self-reported history of hypertension. DM was identified as FBG ≥ 5.60 mmol/L and/or a self-reported history of diabetes.

### Mortality

All-cause mortality was rigorously confirmed as the primary outcome (21). The follow-up duration (in months) was calculated from the date of the baseline interview to the date of death or the last follow-up, with endpoints including survival status. Follow-up visits were primarily conducted by interviews with study participants, their family members, and other contacts to obtain follow-up information. Data on mortality was obtained and validated from official death certificates when available; otherwise, interviews with the next-of-kin or local residential committees were conducted to ensure accurate and reliable mortality data (21, 22).

### Statistical analysis

In our analysis, participants were categorized into 4-level joint variable groups based on a composite of their plasma albumin levels (normal plasma albumin versus hypoproteinemia) and cognitive function status (normal cognition or cognitive impairment), yielding groups: Normal plasma albumin and Normal Cognition, Hypoproteinemia and Normal Cognition, Normal plasma albumin and cognitive impairment, Hypoproteinemia and cognitive impairment. To examine survival disparities among these groups, Kaplan-Meier survival analyses were conducted, with significance assessed via the log-rank test (27).

Data are presented as mean ± standard deviation (SD) or median (interquartile range) for continuous variables and as frequency or counts (%) for categorical variables. For the analysis of baseline characteristics analysis, the statistical differences among the 4-level joint variable groups were tested using ANOVA-tests or Mann–Whitney H-test for continuous variables, and Chi-square tests or Fisher’s exact test for categorical variables. In the combined analysis, three weighted Cox proportional hazards models were used to evaluate the association of albumin, and cognitive function with all-cause mortality, and the Hazard ratios (HRs) and 95% confidence intervals (CIs) were computed and documented. Model 1 was adjusted for age and gender. Model 2 was further adjusted for ethnicity, residence, marital status, education, current smoking, current alcohol drinking, current exercise, BMI, central obesity, and ADL disability. Model 3 was fully adjusted, and adjusted for the confounders in model 2, such as hypertension, diabetes mellitus, heart disease, cerebrovascular disease, respiratory disease, and arthritis. TG, TC, HDL-C, LDL-C, FBG, SUA, and hs-CRP. We also fitted a stratified model to evaluate the association of albumin levels on the outcomes within strata of cognitive function, and a cross-product interaction term of hypoalbuminemia and CI was added to those models above to evaluate whether there was a multiplicative interaction effect on mortality. We used restricted cubic splines to analyze the association of albumin levels with mortality in the total population and individuals with or without CI based on the fully adjusted model. The likelihood ratio test was used to test for potential non-linearity (28).

To estimate the different effects of variables on the results, we conducted subgroup and sensitivity analyses by implementing the following strategies. Subgroup analyses were performed based on key factors, including age, gender, and residence. Sensitivity analyses Including five conditions: (1) We excluded individuals who passed away within 1 year of baseline to avoid the confounding of mortality due to preexisting disease. (2) We ran the models without adjusting for several chronic diseases to prevent unfavorable over-adjusted estimates. (3) Participants who has dementia and cerebrovascular disease were excluded. (4) Participants with MMSE<10 were excluded. The missing individuals in the follow-up (*n* = 331) were divided into two groups for separate processing: (5) Worst-case scenario group: assuming that all individuals lost to follow-up are deceased. (6) Best-case scenario group: Assuming that all individuals lost to follow-up are alive.

Those rigorous measures enhance the robustness of our findings and contribute to a more nuanced understanding of the variables’ effects on the results. Statistical analysis was performed with R software version 3.3.3 (R 205 Project for Statistical Computing). A two-sided *p* < 0.05 was applied to determine statistical significance.

## Results

### Baseline characteristics of participants

Among the 1858 individuals at baseline, 55.7% were female (1,035/1858). The mean age was 86.43 (SD 12.26) years. The median MMSE score was 27 (IQR 18–29), with a corresponding CI prevalence of 24.1%. The mean albumin was 40.02 (SD 4.97), and 12.05% (224/1858) of the participants exhibited hypoproteinemia.

Compared with individuals with normal plasma albumin and normal cognition, those with hypoproteinemia and CI were notably older and presented higher levels of hs-CRP, and lower albumin, MMSE, BMI, TG, TC, SUA. They were more likely to be female, reside in rural areas, experience ADL disability, and less likely to engage in current exercise or display central obesity (all *p* < 0.001). Significant differences were observed in the prevalence of hypertension, DM, heart disease, cerebrovascular diseases, respiratory diseases, and arthritis among the groups. The baseline characteristics of the four-level joint albumin /cognition function groups were presented in Table 1.

**Table 1:** Characteristics of the study population according to plasma albumin and cognitive function status.

	Normal plasma albumin and normal cognition (*n* = 1,288)	Hypoproteinemia and normal cognition (*n* = 123)	Normal plasma albumin and CI (*n* = 346)	Hypoproteinemia and CI (*n* = 101)	Total (*n* = 1858)	*p*-value
Age, mean (SD)	82.06 ± 11.61	89.01 ± 11.13	96.82 ± 7.46	97.86 ± 7.62	86.43 ± 12.26	<0.001
Albumin, mean (SD)	41.62 ± 3.90	31.92 ± 2.57	39.59 ± 3.58	30.85 ± 3.43	40.02 ± 4.97	<0.001
MMSE, median (IQR)	28 (26–29)	27 (24–29)	7 (4–14)	6 (0–10)	27 (18–29)	<0.001
BMI, mean (SD)	21.62172 ± 3.93	20.28 ± 3.96	20.07 ± 4.19	19.30 ± 4.26	21.17 ± 4.06	<0.001
TG, mean (SD)	1.03 ± 0.65	0.89 ± 0.60	0.91 ± 0.44	0.68 ± 0.37	0.98 ± 0.63	<0.001
TC, mean (SD)	4.42 ± 0.93	3.49 ± 0.89	4.27 ± 0.97	3.49 ± 0.84	4.28 ± 0.97	<0.001
HDL-C, mean (SD)	1.33 ± 0.36	1.04 ± 0.28	1.29 ± 0.33	1.11 ± 0.26	1.29 ± 0.36	<0.001
LDL-C, mean (SD)	2.62 ± 0.79	2.05 ± 0.77	2.57 ± 0.83	2.07 ± 0.70	2.54 ± 0.81	<0.001
FBG, mean (SD)	4.58 ± 2.16	5.08 ± 3.63	4.77 ± 1.57	4.77 ± 1.44	4.66 ± 2.16	0.057
hs-CRP, median (IQR)	0.83 (0.39–2.08)	1.28 (0.40–5.45)	1.03 (0.35–3.07)	1.76 (0.65–7.46)	0.91 (0.39–2.51)	
SUA, mean (SD)	296.63 ± 90.30	278.08 ± 75.24	273.21 ± 83.00	254.91 ± 92.14	289.63 ± 88.57	<0.001
Gender, *n* (%)						<0.001
Male	664 (51.6)	59 (48.0)	80 (23.1)	20 (19.8)	823 (44.3)	
Female	624 (48.4)	64 (52.0)	266 (76.9)	81 (80.2)	1,035 (55.7)	
Ethnicity, *n* (%)						0.003
Han	1,193 (92.6)	102 (82.9)	319 (92.2)	93 (92.1)	1,707 (91.9)	
Others	95 (7.4)	21 (17.1)	27 (7.8)	8 (7.9)	151 (8.1)	
Residence, *n* (%)						0.010
Urban	227 (17.6)	15 (12.2)	62 (17.9)	6 (5.9)	310 (16.7)	
Rural	1,061 (82.4)	108 (87.8)	284 (82.1)	95 (94.1)	1,548 (83.3)	
Marital status, *n* (%)						<0.001
Married and living with spouse	605 (47.0)	39 (31.7)	28 (8.1)	9 (8.9)	681 (36.7)	
Other	683 (53.0)	84 (68.3)	318 (91.9)	92 (91.1)	1,177 (63.3)	
Education levels, *n* (%)						<0.001
Illiteracy (0 year)	735 (57.1)	69 (56.1)	309 (89.3)	88 (87.1)	1,201 (64.6)	
Primary (1–6 years)	415 (32.2)	50 (40.7)	33 (9.5)	12 (11.9)	510 (27.4)	
Middle and advanced (>6 years)	138 (10.7)	4 (3.3)	4 (1.2)	1 (1.0)	147 (7.9)	
Current smoking, *n* (%)	244 (18.9)	26 (21.1)	23 (6.6)	7 (6.9)	300 (16.1)	<0.001
Current alcohol drinking, *n* (%)	404 (31.4)	38 (30.9)	76 (22.0)	20 (19.8)	538 (29.0)	0.001
Current exercise, *n* (%)	213 (16.5)	17 (13.8)	23 (6.6)	4 (4.0)	257 (13.8)	<0.001
Central obesity, *n* (%)	535 (41.5)	37 (30.1)	112 (32.4)	24 (23.8)	708 (38.1)	<0.001
ADL disability, *n* (%)	111 (8.6)	26 (21.1)	196 (56.6)	63 (62.4)	396 (21.3)	<0.001
Hypertension, *n* (%)	993 (77.1)	80 (65.0)	243 (70.2)	59 (58.4)	1,375 (74.0)	<0.001
Diabetes mellitus, *n* (%)	88 (6.8)	17 (13.8)	32 (9.2)	9 (8.9)	146 (7.9)	0.029
Heart disease, *n* (%)	105 (8.2)	3 (2.4)	26 (7.5)	7 (6.9)	141 (7.6)	0.002
Cerebrovascular disease, *n* (%)	94 (7.3)	3 (2.4)	40 (11.6)	11 (10.9)	148 (8.0)	<0.001
Respiratory disease, *n* (%)	111 (8.6)	12 (9.8)	29 (8.4)	4 (4.0)	156 (8.4)	0.003
Arthritis *n* (%)	135 (10.5)	16 (13.0)	27 (7.8)	9 (8.9)	187 (10.1)	<0.001
Dementia	6 (0.5)	2 (1.6)	16 (4.6)	10 (9.9)	34 (1.8)	<0.001

BMI, body mass index; TG, triglycerides; TC, total cholesterol; HDL-C, high-density lipoprotein cholesterol; LDL-C, low-density lipoprotein cholesterol; FBG, fasting blood glucose; SUA, serum uric acid; hs-CRP, high sensitivity c-reactive protein; CI, cognitive impairment; ADL, activity of daily living.

### Individual associations of albumin and cognitive status with mortality

Table 2 illustrated the distinct associations of plasma albumin levels, cognition status with all-cause mortality. Upon multivariate adjustment (Model 3), a significant, monotonic, and positive association was observed between CI and increased mortality, with a HR of 1.69 (95%CI, 1.43, 1.99). Similarly, hypoproteinemia also exhibited a significant association with increased mortality in the fully adjusted model (HR = 1.33, 95%CI: 1.10, 1.62). Consistent and statistically significant results were observed when a Per-SD increase was considered, indicating a reduced risk of all-cause mortality in both MMSE scores (HR = 0.78, 95%CI: 0.73, 0.84) and albumin levels (HR = 0.85, 95%CI, 0.78, 0.93). Notably, individuals in the highest quartile of albumin levels showed a 34% decreased risk of mortality compared to those in the lowest quartile (HR Q4 *vs* Q1: 0.66, 95%CI: 0.54, 0.81) in the adjusted model with age, gender. Furthermore, even after further adjustment for demographic factors, lifestyle, physical measurement indicators, chronic conditions, and fasting blood specimens (Model 3), with only a modest attenuation (HR Q4 *vs* Q1: 0.72, 95%CI, 0.56, 0.92). The trend across quartiles remained statistically significant (*p*-trend<0.001, Table 2).

**Table 2:** Hazard ratios for the individual associations of albumin levels and cognitive function status with all-cause mortality (*N* = 1858).

Exposure variables	No. of death	Crude model	Model 1	Model 2	Model 3
		HR (95%CI)	HR (95%CI)	HR (95%CI)	HR (95%CI)
Cognition groups					
Normal cognition (*n* = 1,411)	530	1 (Ref)	1 (Ref)	1 (Ref)	1 (Ref)
Cognitive impairment (*n* = 447)	391	4.50 (3.94, 5.14)*	1.97 (1.70, 2.30)*	1.58 (1.33, 1.88)*	1.69 (1.43, 1.99)*
Per-SD increase (MMSE score)		0.52 (0.49, 0.54)*	0.73 (0.68, 0.78)*	0.78 (0.73, 0.84)*	0.78 (0.73, 0.84)*
Albumin					
Normal Albumin (≥35 g/L) (*n* = 1,634)	752	1 (Ref)	1 (Ref)	1 (Ref)	1 (Ref)
Hypoproteinemia (<35 g/L) (*n* = 224)	169	2.35 (1.99, 2.78)*	1.46 (1.24, 1.74)*	1.48 (1.22, 1.78)*	1.33 (1.10, 1.62)**
Per-SD increase		0.65 (0.61, 0.70)*	0.84 (0.78, 0.90)*	0.86 (0.80, 0.92)*	0.85 (0.78, 0.93)*
Albumin cut-offs by quartiles					
Q1 (<39.1 g/L) (*n* = 467)	319	1 (Ref)	1 (Ref)	1 (Ref)	1 (Ref)
Q2 (39.1–42.8 g/L) (*n* = 482)	267	0.66 (0.56, 0.78)*	0.82 (0.70, 0.97)***	0.84 (0.70, 0.99)***	0.87 (0.73, 1.03)
Q3 (42.9–47.6 g/L) (*n* = 473)	191	0.44 (0.37, 0.53)*	0.70 (0.58, 0.84)*	0.70 (0.58, 0.86)**	0.76 (0.62, 0.94)***
Q4 (≥47.6 g/L) (*n* = 436)	144	0.34 (0.28, 0.41)*	0.66 (0.54, 0.81)*	0.71 (0.57, 0.89)**	0.72 (0.56, 0.92)**
*p* for trend		<0.001	<0.001	<0.001	0.003

CIs indicates confidence intervals; Hazard ratio (95%CI) was calculated from Cox models. * *p*-value < 0.001; ** *p*-value < 0.01; *** *p*-value < 0.05.

Crude model was an unadjusted model.

Model 1 was adjusted with age, gender.

Model 2 was adjusted for age, gender, ethnicity, residence, marital status, education, current smoking, current alcohol drinking, current exercise, BMI, central obesity, ADL disability.

Model 3 was fully adjusted model: adjusted for the confounders in model 2, such as hypertension, diabetes mellitus, heart disease, cerebrovascular disease, respiratory disease, and arthritis. TG,TC, HDL-C, LDL-C, FBG, SUA, and hs-CRP.

### Combined associations of albumin and cognitive status on mortality

**Figure 1:**
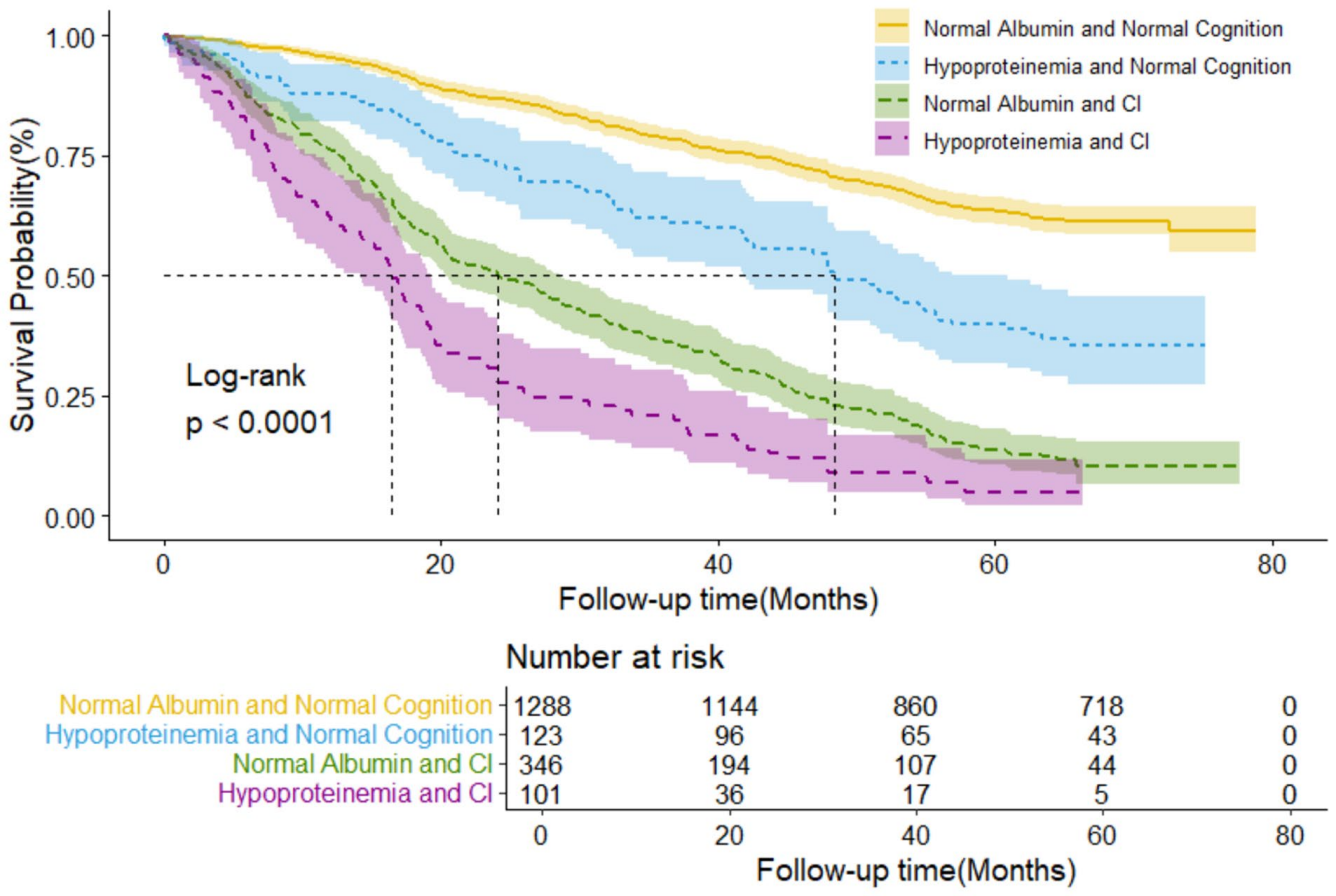
Kaplan-Meier survival curves by the 4-level joint albumin level/cognition function groups in older adults (*N* = 1,858). Log-rank *p* < 0.000.

Figure 1 illustrated the Kaplan-Meier survival curves for four-level joint variable groups of participants, revealing compelling differences in survival patterns (log-rank test *p* < 0.001). The median follow-up periods for all-cause mortality varied across the four albumin/cognition function groups: 64.07, 42.28, 22.26, and 16.43 months, respectively. During an unweighted median follow-up period of 48.85 months (IQR, 22.63–64.86 months), 921 deaths were recorded.

Table 3 comprehensively outlined the combined associations between albumin and cognitive function on all-cause mortality. Specifically, we observed monotonic and positive associations indicating that as we move from normal albumin and normal cognition to combinations where either or both factors are compromised, the mortality risk increases. Notably, the groups with hypoproteinemia and CI exhibited the most pronounced increase in mortality risk (*p*-trend <0.001 for all comparisons, as shown in Table 3). Compared to individuals with normal albumin and normal cognition, those with hypoproteinemia and CI had the highest mortality risk, with a HR of 2.55 (95%CI: 1.96, 3.32). Furthermore, the mortality risk increased by 57% among participants with CI and normal albumin (HR = 1.57, 95%CI: 1.30, 1.89). However, the significant association between hypoproteinemia, normal cognition, and mortality was no longer evident (HR = 1.11, 95%CI: 0.83, 1.47) after adjusting for confounding variables in Model 3. Furthermore, the joint analysis revealed a noteworthy interaction between albumin and cognitive status in predicting 6-year mortality (*p* for interaction<0.001, Supplementary Table S1).

**Table 3:** Hazard ratios for the combined associations of plasma albumin and cognitive impairment status with all-cause mortality (*N* = 1858).

Groups	No. of death	Crude model^†^	Model 1^†^	Model 2^†^	Model 3^†^
		HR (95%CI)	HR (95%CI)	HR (95%CI)	HR (95%CI)
Normal albumin and normal cognition (*n* = 1,288)	457	1 (Ref)	1 (Ref)	1 (Ref)	1 (Ref)
Hypoproteinemia and normal cognition (*n* = 123)	73	2.06 (1.61, 2.64)*	1.34 (1.04, 1.72)**	1.29 (0.99, 1.69)	1.11 (0.83, 1.47)
Normal albumin and cognitive impairment (*n* = 346)	295	4.42 (3.81, 5.13)*	1.90 (1.61, 2.25)*	1.49 (1.23, 1.80)*	1.57 (1.30, 1.89)*
Hypoproteinemia and cognitive impairment (*n* = 101)	96	7.09 (5.67, 8.86)*	2.93 (2.31, 3.71)*	2.47 (1.89, 3.23)*	2.55 (1.96, 3.32)*
*p* for trend		<0.001	<0.001	<0.001	<0.001

CIs indicates confidence intervals; Hazard ratio (95%CI) was calculated from Cox models. * *p*-value < 0.001;** *p*-value < 0.05. † *p* for interaction (Cognitive function status ∗ plasma albumin category) < 0.001.

Crude model was an unadjusted model.

Model 1 was adjusted with age, gender.

Model 2 was adjusted for age, gender, ethnicity, residence, marital status, education, current smoking, current alcohol drinking, current exercise, BMI, central obesity, ADL disability.

Model 3 was fully adjusted model: adjusted for the confounders in model 2, such as hypertension, diabetes mellitus, heart disease, cerebrovascular disease, respiratory disease, and arthritis. TG,TC, HDL-C, LDL-C, FBG, SUA, and hs-CRP.

**Figure 2:**
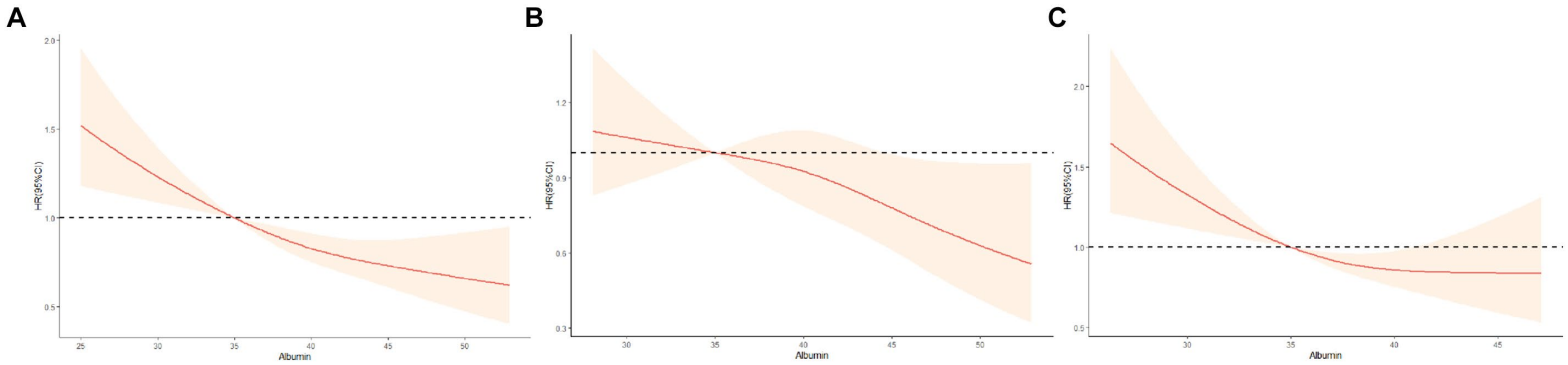
Restricted cubic splines for the association of albumin level with all-cause mortality according to cognitive function status. **(A)** All participants; **(B)** Non-cognitive impairment group; **(C)** Cognitive impairment group. Hazard ratios (HRs) are indicated by red solid lines and 95%CIs by shaded areas. All models were adjusted for age, gender, ethnicity, residence, marital status, education, current smoking, current alcohol drinking, current exercise, BMI, central obesity, ADL disability, hypertension, diabetes mellitus, heart disease, cerebrovascular disease, respiratory disease, and arthritis. TG,TC, HDL-C, LDL-C, FBG, SUA, and hs-CRP.

The Figure 2 illustrates the restricted cubic splines of HRs for plasma albumin level as continuous variables. In the total population, a near reverse J-shaped relationship between albumin levels and mortality was observed (*p* for non-linearity>0.05, Figure 2A). This relationship was significantly influenced by cognitive function status, with all *p* values for non-linearity exceeding 0.05, indicating a modification of the curve shape by cognitive function (Figure 2A). For participants without CI, there was a steep decrease in the risk of mortality with increasing albumin level when albumin≥35 g/L. However, we found that the relationship was flattened in participants with normal cognition when albumin <35 g/L (Figure 2B). For participants with CI, the risk of all-cause mortality increased sharply when albumin level < 35 g/L, and was higher than the total population and participants without CI. However, all-cause mortality was slowly decreased when albumin≥35 g/L. And a inversely but not significant association was noted for concentrations above 45 g/L (Figure 2C).

### Stratified results and sensitivity analyses

**Figure 3:**
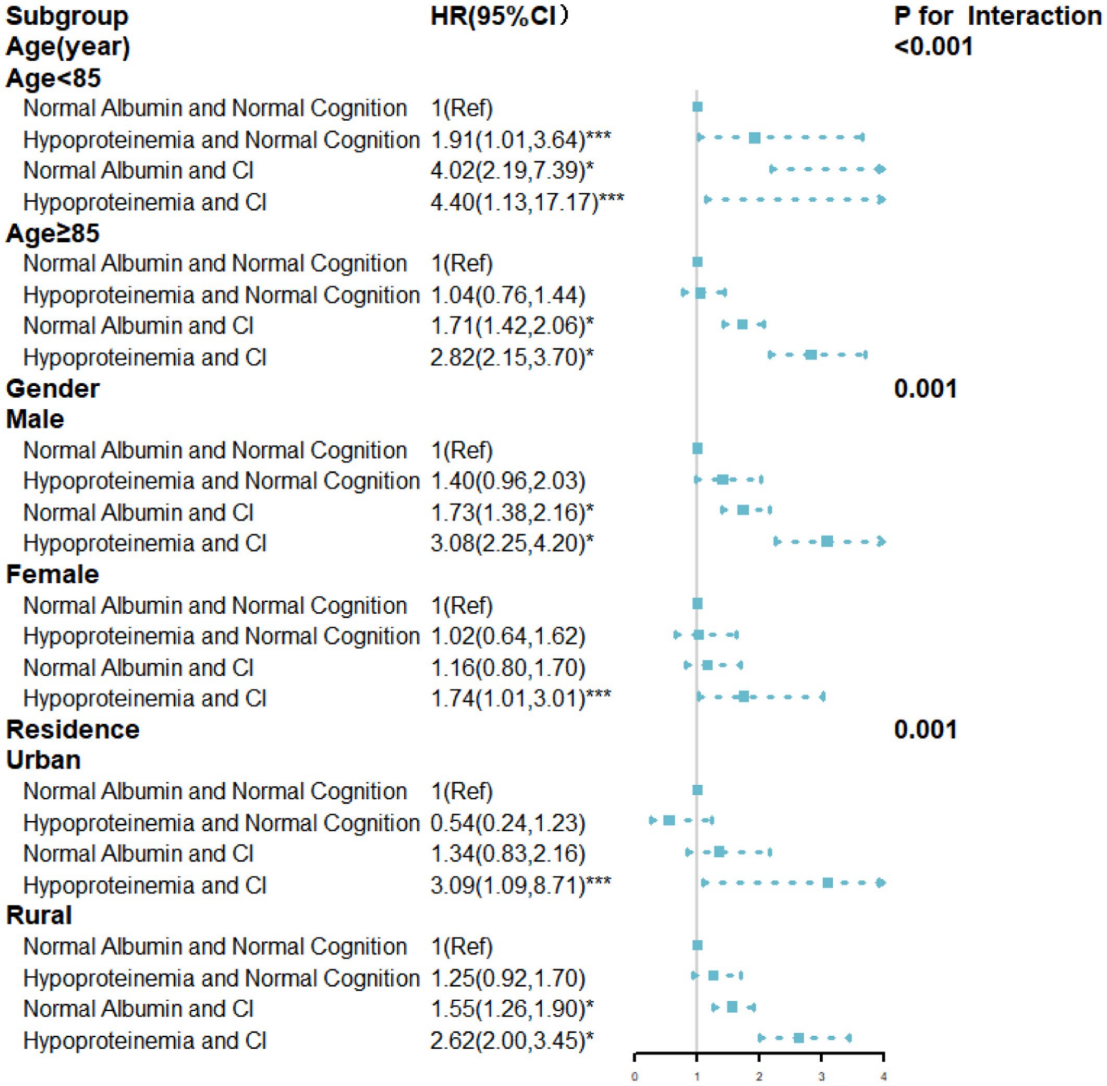
HRs for the combined associations of cognition function and albumin with all-cause mortality according to the classification of age, gender, residence. All models were adjusted for age (not in the age subgroup), gender (not in the gender subgroup), and residence (not in the residence subgroup), ethnicity, marital status, education, current smoking, current alcohol drinking, current exercise, BMI, central obesity, ADL disability, hypertension, diabetes mellitus, heart disease, cerebrovascular disease, respiratory disease, and arthritis. TG,TC, HDL-C, LDL-C, FBG, SUA, and hs-CRP. * *p*-value<0.001; ** *p*-value<0.01; *** *p*-value<0.05.

The stratified results according to age, gender, and residence were presented in Figure 3. Among older adults aged <85 years, the hypoproteinemia and CI group had a higher risk of mortality (HR = 4.40, 95%CI: 1.13, 17.17). Similar trends were observed in those aged ≥85 years (HR = 2.82, 95%CI: 2.15, 3.70), (*p* for interaction<0.001). Furthermore, the association between albumin, cognitive function, and mortality manifested gender-specific differences. In males, individuals with hypoproteinemia and CI faced a significantly elevated mortality risk, with the impact being more pronounced in males than females (*p* for interaction = 0.001). Among urban older adults, only those with hypoproteinemia and CI had a significantly higher mortality risk (HR = 3.09, 95%CI: 1.09–8.71) compared to those with normal albumin and cognition. In rural areas, individuals with hypoproteinemia and CI also had a significantly higher mortality risk (HR = 2.62, 95%CI: 2.00, 3.45). Additionally, rural individuals with normal albumin and CI had a higher mortality risk (HR = 1.55, 95%CI: 1.26, 1.90), (*p* for interaction<0.001).

Regarding sensitivity analyses, similar results emerged among individuals who died within 1 year of the baseline date (HR = 2.55, 95%CI: 1.95, 3.33). Rerunning the models without adjustment for chronic diseases, the effect of albumin and cognitive impairment on all-cause (HR = 2.64, 95%CI: 2.04, 3.42) mortality risk was consistent with the primary outcome. Even after excluding individuals with dementia, cerebrovascular disease (including stroke or CVD), or MMSE scores below 10, the results remained robust. Additionally, for the 113 individuals lost to follow-up, we simulated the impact of missing data using two extreme scenarios. Through sensitivity analysis with these scenarios, we observed that our study results remained robust even under the most extreme assumptions (Supplementary Table S2).

## Discussion

In our study, which encompassed 1,858 Chinese older adults aged 65 and above, we established a significant and independent association between hypoalbuminemia (<35 g/L) and CI, with each factor being identified as an independent predictor of all-cause mortality. However, the group of hypoalbuminemia and normal cognition not retain statistical significance in the fully adjusted model. This finding is consistent with existing literature that individually associates hypoproteinemia and CI with an increased risk of mortality among older adults. Importantly, our study further investigates the combined effect of hypoproteinemia and CI on mortality risk, revealing a synergistic relationship that amplifies the overall risk of death in the elderly. Noteworthy is the observation that individuals experiencing both conditions concurrently face the highest mortality risk, underscoring the importance of jointly considering these factors in geriatric risk assessment.

We found that the association between albumin concentration, cognitive status, and all-cause mortality rates in individuals is consistent with previous studies. Even after adjusting for demographic factors, lifestyle, BMI, and chronic diseases, hypoalbuminemia and CI may still be related to mortality risk. This information can be utilized for diagnosing CI and may contribute to the prevention of this condition. This study is the first to discover an interaction between plasma albumin, cognitive function, and all-cause mortality in the elderly population and among men in China. It can be used to predict the 6-year mortality rate (*p* for interaction <0.001), indicating a joint association of hypoalbuminemia and CI with mortality. In a study on longitudinal aging conducted in Amsterdam, among 713 respondents, after adjusting for potential confounding factors, higher levels of albumin were associated with a lower risk of cardiovascular disease (RR 0.88, 95%CI: 0.79, 0.98). However, this study indicated that albumin exposure was not associated with mortality outcomes (29). A study found that in 150 elderly patients undergoing transcatheter aortic valve implantation, the 2.1-year mortality rate (35%) in the low albumin group (with a critical value of 40 g/L) was higher than that in the normal group (19%), with a *p*-value of 0.01. Multivariate analysis showed that preoperative low albumin was independently associated with more than a two-fold increase in the 2.1-year all-cause mortality rate (HR = 2.28, 95%CI: 1.17–4.44). Postoperative low albumin was also strongly associated with all-cause mortality rate (HR = 2.47, 95%CI: 1.28–4.78). Virginie Lam and colleagues found a significant positive correlation between cognitive performance and plasma albumin homeostasis in 222 healthy participants (143 females) aged 43–84 years (average age 65 years). However, both studies did not consider the impact of albumin and cognitive function on mortality rate (30).

To our knowledge, this is the first cohort study to investigate the comprehensive effects of plasma albumin and cognition on mortality in Chinese older adults. Several previous studies have indicated a relationship between albumin and mortality (31–34). However, our study found that the significant association between hypoalbuminemia and mortality disappeared under the premise of normal cognitive function (HR = 1.11. 95%CI, 0.83–1.47), This can be explained by the following factors: Firstly, in the unadjusted model, hypoalbuminemia might be associated with other health issues, thereby significantly increasing the risk of mortality. However, after adjusting for these health problems, the independent effect of hypoalbuminemia on mortality risk was diminished. Pure hypoalbuminemia might represent an early or milder stage of the disease, which may not yet have a severe impact on the mortality of the patients. Secondly, due to the limited sample size of the hypoalbuminemia and normal cognition group, which included only 123 patients, increasing the number of covariates could lead to a wider HR and 95%CI, thereby resulting in a loss of statistical significance. In our study, low albumin levels/cognitive combination variables are more sensitive in predicting all-cause mortality in men and younger elderly individuals (aged 65–85). Our research focuses on elderly people in China; therefore, this association is worth further investigation in other races and age groups. Notably, this study bridges a gap by systematically investigating the interrelationship between these two factors and their joint impact on all-cause mortality.

We found an reverse J-shaped association between albumin levels and mortality. This suggests that for normal cognition participants, elevated albumin levels resulted in a significantly lower risk of mortality. However, in participants with hypoproteinemia, the relationship between cognitive normalcy and mortality leveled off. For participants with cognitive dysfunction, hypoproteinemia resulted in a significantly increased risk of all-cause mortality. For participants with cognitive dysfunction, normal albumin levels resulted in a progressively lower risk of all-cause mortality. Several pathophysiologic mechanisms may help explain our results. Potential additional or combined effects of albumin levels and cognitive impairment on the risk of death in older adults, malnutrition, inflammation, and oxidative reactions may be common factors mediating the observed associations in this study. Firstly, in older adults, malnutrition, either due to intrinsic nutritional deficiency or age-related diseases, may lead to a decrease in albumin levels, which is considered one of the main indicators of nutritional status in patients (35, 36). Malnutrition may impair cognitive function through the inhibition of neuronal plasticity and the occurrence of white matter hyperintensities (37). Secondly, Hillmer et al. found that albumin is still controversial as a nutritional indicator for the elderly, but it can be used as one of the inflammatory response indicators (38–40). Increasing evidence indicates that the inflammatory mechanism is involved in the pathogenesis of cognitive impairment (41). Albumin is a negative acute-phase protein, and inflammatory factors may decrease plasma concentration of albumin in patients with chronic diseases by inhibiting albumin synthesis, accelerating protein denaturation and increasing vascular permeability (42, 43). In older adults, genetic factors, unhealthy lifestyles, and the impacts of diseases increase inflammatory activity. In this situation, individuals with low levels of albumin and comprehensive risk indicators for CI may have higher levels of inflammation, resulting in a greater risk of mortality compared to individuals with elevation in a single component. At the same time, a decrease in the body’s albumin content leads to an imbalance between oxidation and antioxidant capacity within the body, accelerating oxidative damage to nerve cells and the development of cognitive impairments (44). There is strong evidence that persistent CI is accompanied by a persistent inflammatory response and a state of endothelial dysfunction, and that increasing albumin levels significantly improves cognitive dysfunction and quality of life, which exerts a neuroprotective effect primarily by decreasing endothelial dysfunction and systemic inflammation, thus reducing mortality (45, 46). These may be why the elderly with normal albumin levels showed better cognitive performance and lower mortality than with hypoproteinemia.

Our study’s strengths are multifaceted. Firstly, our study’s strengths lie in its comprehensive nature, leveraging data from the CLHLS, ensuring a robust foundation for the findings. Secondly, the large sample size, rigorous follow-up procedures, and meticulous adjustments for confounding variables enhance the result credibility. Thirdly, to the best of our knowledge, there are no published studies on the association between plasma albumin and cognitive status on mortality risk in older Chinese adults. The novel aspect of this study lies in its comprehensive approach, integrating robust data from the CLHLS in 2012 and employing advanced statistical methodologies, such as Cox proportional hazards regression models and restricted cubic splines. The utilization of these statistical methods allows for a nuanced analysis of the associations, offering valuable insights into the dose–response relationship between albumin levels and mortality risk, as well as the complex interplay between cognitive impairment and plasma albumin concentrations.

The present study, while informative, has several noteworthy limitations that warrant discussion. Firstly, our research focuses solely on a single blood indicator, plasma albumin. Combining multiple indicators may enhance the predictive accuracy of mortality rates (47), implying that our results may underestimate the association between plasma albumin and mortality rates. Secondly, although we attempted to adjust for potential confounding factors, the reliance on self-reported demographic characteristics and lifestyle information may introduce reporting bias. Thirdly, the absence of detailed economic and healthcare data, including specific economic status and access to medical services, may influence our mortality findings and represents an additional limitation. Furthermore, the exclusion of comprehensive nutritional status and dietary intake information, which could significantly affect our results, is another shortcoming. Fourthly, the study is based solely on baseline measurements of plasma albumin and cognitive function, which may change over the follow-up period. The absence of repeated assessments of plasma albumin and cognitive function during follow-up, preventing an exploration of age-related changes in these factors over time. Understanding these longitudinal changes is critical for a comprehensive assessment of their impact on mortality. In future research, we aim to incorporate longitudinal assessments of plasma albumin and cognitive function to better understand their dynamic interactions and long-term effects on mortality. Lastly, by focusing exclusively on samples from longevity villages, our study limits the external validity of the results. The need for further research to validate these findings across various cohorts is evident, as the association between hypoproteinemia and CI on mortality risk has not been extensively studied in other populations. This limitation underscores the importance of cautious interpretation of our results and the necessity for additional research to confirm their applicability to broader populations.

Given the significant association between plasma albumin levels, CI and mortality in Chinese elderly, these findings underscore the urgent need for targeted policy and clinical responses. It is imperative for policymakers to focus on healthcare infrastructure that caters to the aging demographic’s unique requirements. This includes improving access to healthcare services, particularly in rural areas, and bolstering specialized geriatric care. Furthermore, the establishment of community-based nutritional support programs, complemented by public health campaigns to increase awareness of the importance of cognitive function and nutrition. In clinical practice, healthcare providers should integrate routine cognitive and nutritional status assessments into the care of older adults to facilitate early detection and intervention. A multidisciplinary approach, including geriatricians, neurologists, and nutritionists, is crucial for providing comprehensive care to patients with CI. In addition, personalized programs, such as nutritional support and cognitive stimulation, are vital for managing cognitive decline. Education for patients and their families regarding the risks, prevention, and management of CI and nutrition is paramount. It enhances treatment adherence and life quality, ensuring that individuals are empowered to manage their health. Moreover, ongoing monitoring of albumin levels and cognitive function, with care plan adjustments, is foundational for effective health management, ensuring care adapts to the elderly’s evolving needs. By incorporating these implications into policy and clinical practice, we can not only proactively address the health challenges faced by the Chinese elderly but also enhance individual health outcomes.

## Conclusion

This study provides valuable insights into the intricate relationship between plasma albumin, CI, and the combined effect on all-cause mortality among Chinese community-dwelling older adults. Notably, the combined effect in predicting all-cause mortality was stronger for older male, younger seniors and those living in rural areas. Understanding the nuances of this association is crucial for tailoring interventions and individualized clinical care for this vulnerable population.

## Data availability statement

The datasets presented in this study can be found in online repositories. The names of the repository/repositories and accession number(s) can be found in the article/Supplementary material.

## Ethics statement

The studies involving humans were approved by Research Ethics Committee of both Duke University and Peking University (IRB00001052-13074). The studies were conducted in accordance with the local legislation and institutional requirements. The participants provided their written informed consent to participate in this study.

## Author contributions

Z-qL: Conceptualization, Methodology, Software, Writing – original draft, Writing – review & editing. X-xL: Methodology, Visualization, Writing – original draft. X-fW: Data curation, Investigation, Methodology, Supervision, Writing – original draft. CS: Methodology, Supervision, Visualization, Writing – original draft. FC: Data curation, Methodology, Supervision, Visualization, Writing – original draft. X-mG: Supervision, Writing – original draft. YZ: Data curation, Investigation, Methodology, Writing – review & editing. J-pL: Conceptualization, Funding acquisition, Methodology, Supervision, Writing – review & editing.
