# Supplementary material for: Synergistic impact of plasma albumin and cognitive function on all-cause mortality in Chinese older adults: a prospective cohort study
Source: Front Nutr. 2024 Jul 24;11:1410196. doi: 10.3389/fnut.2024.1410196 (PMC11303755; doi:10.3389/fnut.2024.1410196)
Supplement: Supplementary file 1 [file Table_1.docx]

Synergistic Impact of Plasma Albumin and Cognitive Function on All-Cause Mortality in Older Adults

Supplementary Table 1. Hazard ratios for the association between albumin and all-cause mortality by cognition impairment or not.

Supplementary Table 2. Sensitive of the combined associations of albumin and cognitive impairment status with all-cause mortality

Supplementary Figure 1.The flow chart of the study.

| Supplementary Table 1. Hazard ratios for the association between albumin and all-cause mortality by cognition impairment or not. | | | | | |
| --- | --- | --- | --- | --- | --- |
| Exposure variables | No. of Death | Crude model | Model 1 | Model 2 | Model 3 |
|  |  | HR (95% CI) | HR (95% CI) | HR (95% CI) | HR (95% CI) |
| Normal cognition(N=1411) |  |  |  |  |  |
| Normal Albumin(≥35 g/L)(n=1288)  Hypoproteinemia(<35 g/L)(n=123) | 457  73 | 1(Ref)  2.07(1.62,2.65)*** | 1(Ref)  1.239(0.96,1.59) | 1(Ref)  1.16(0.90,1.51) | 1(Ref)  1.06(0.78,1.44) |
| Cognitive impairment(N=447) |  |  |  |  |  |
| Normal Albumin(≥35 g/L)(n=346)  Hypoproteinemia(<35 g/L)(n=101) | 295  96 | 1(Ref)  1.58(1.25,1.99)*** | 1(Ref)  1.55(1.23,1.95)*** | 1(Ref)  1.72(1.35,2.19)*** | 1(Ref)  1.61(1.23,2.10)*** |
| *P for interaction* |  | <0.001 | <0.001 | <0.001 | <0.001 |
| Note: CIs indicates confidence intervals; Hazard ratio (95% CI) was calculated from Cox models. ** P*-value<0.001;*** P*-value<0.01;**** P*-value<0.05  Crude model was an unadjusted model;  Model 1 was adjusted with age, gender;  Model 2 was adjusted for age, gender, ethnicity, residence, marital status, education, current smoking, current alcohol drinking, current exercise, BMI, central obesity, ADL disability;  Model 3 was fully adjusted model: adjusted for the confounders in model 2, such as hypertension, diabetes mellitus, heart disease, cerebrovascular disease, respiratory disease, and arthritis. TG,TC, HDL-C, LDL-C, FBG, SUA, and hs-CRP. | | | | | |

| **Supplementary Table 2. Sensitive of the combined associations of albumin and cognitive impairment status with all-cause mortality** | | | | | |
| --- | --- | --- | --- | --- | --- |
| Groups | No. of Death | Crude model | Model 2 | Model 3 | Model 4 |
|  |  | HR (95% CI) | HR (95% CI) | HR (95% CI) | HR (95% CI) |
| **Sensitive 1:Excluding the participants who died in the first year (N = 1844 )** | | | | | |
| Normal Albumin and Normal Cognition(*n*=1285)  Hypoproteinemia and Normal Cognition(*n*=121)  Normal Albumin and CI(*n*=340)  Hypoproteinemia and CI(*n*=98) | 454  71  289  93 | 1(Ref)  2.02(1.58,2.60)***  4.39(3.78,5.11)***  7.03(5.60,8.82)*** | 1(Ref)  1.31(1.02,1.69)*****  1.88(1.59,2.23)***  2.89(2.27,3.68)*** | 1(Ref)  1.24(0.96,1.60)  1.60(1.33,1.91)***  2.65(2.06,3.40)*** | 1(Ref)  110(0.82,1.47)  1.55(1.29,1.87)***  2.55(1.95,3.33)*** |
| **Sensitive 2: Reran the models without adjustment for chronic diseases (N = 1858 )** | | | | | |
| Normal Albumin and Normal Cognition(*n*=1288)  Hypoproteinemia and Normal Cognition(*n*=123)  Normal Albumin and CI(*n*=346)  Hypoproteinemia and CI(*n*=101) | 457  73  295  96 | 1(Ref)  2.06(1.61,2.64)***  4.42(3.81,5.13)***  7.09(5.67,8.86)*** | 1(Ref)  1.34(1.04,1.72)****  1.90(1.61,2.25)***  2.93(2.31,3.71)*** | 1(Ref)  1.29(0.99,1.69)  1.49(1.23,1.80)***  2.47(1.89,3.23)*** | 1(Ref)  1.12(0.85,1.47)  1.59(1.33,1.91)***  2.64(2.04,3.42)*** |
| **Sensitive 3: Excluding the participants who has dementia and cerebrovascular disease(N=1831)** | | | | | |
| Normal Albumin and Normal Cognition(*n*=1269)  Hypoproteinemia and Normal Cognition(*n*=118)  Normal Albumin and CI(*n*=343)  Hypoproteinemia and CI(*n*=101) | 449  69  292  96 | 1(Ref)  2.04(1.58,2.63)***  4.41(3.80,5.12)***  7.12(5.69,8.91)*** | 1(Ref)  1.33(1.03,1.73)*****  1.90(1.61,2.26)***  2.95(2.33,3.75)*** | 1(Ref)  1.25(0.96,1.63)  1.62(1.35,1.94)***  2.69(2.10,3.45)*** | 1(Ref)  1.11(0.83,1.47)  1.57(1.31,1.89)***  2.58(1.98,3.35)*** |
| **Sensitive 4: Excluding the participants with MMSE<10 (n=1578)** | | | | | |
| Normal Albumin and Normal Cognition(*n*=1288)  Hypoproteinemia and Normal Cognition(*n*=123)  Normal Albumin and CI(*n*=141)  Hypoproteinemia and CI(*n*=26) | 457  73  117  25 | 1(Ref)  2.08(1.62,2.66)  3.94(3.21,4.84)***  6.58(4.39,9.87)*** | 1(Ref)  1.28(1.00,1.65)  1.79(1.44,2.23)***  2.57(1.70,3.88)*** | 1(Ref)  1.20(.927,1.56)  1.56(1.24,1.96)***  2.62(1.73,3.97)*** | 1(Ref)  1.13(0.84,1.51)  1.50(1.18,1.89)***  2.52(1.65,3.86)*** |
| **Sensitive 5: Add 331 participants who lost to follow-up were included and were defined as alive(n=2189)** | | | | | |
| Normal Albumin and Normal Cognition(*n*=1522)  Hypoproteinemia and Normal Cognition(*n*=158)  Normal Albumin and CI(*n*=387)  Hypoproteinemia and CI(*n*=122) | 457  73  295  96 | 1(Ref)  1.87(1.46,2.39)*  4.40(3.79,5.10)*  5.80(4.64,7.25)* | 1(Ref)  1.20(0.93,1.54)  1.95(1.65,2.30)*  2.49(1.97,3.15)* | 1(Ref)  1.26(0.98,1.63)  1.61(1.34,1.92)*  2.67(2.08,3.41)* | 1(Ref)  1.11(0.83,1.47)  1.57(1.30,1.89)*  2.55(1.96,3.32)* |
| **Sensitive 6: Add 331 participants who were lost to follow-up were included and were defined as death(n=2189)** | | | | | |
| Normal Albumin and Normal Cognition(*n*=1522)  Hypoproteinemia and Normal Cognition(*n*=158)  Normal Albumin and CI(*n*=387)  Hypoproteinemia and CI(*n*=122) | 691  108  336  117 | 1(Ref)  1.80(1.47,2.20)*  3.50(3.07,4.00)*  4.73(3.88,5.77)* | 1(Ref)  1.26(1.03,1.55)*****  1.86(1.60,2.15)*  2.45(1.98,3.02)* | 1(Ref)  1.21(0.97,1.50)  1.60(1.36,1.88)*  2.10(1.69,2.63)* | 1(Ref)  1.09(0.87,1.38)  1.60(1.35,1.90)*  2.13(1.67,2.71)* |
| Note: CIs indicates confidence intervals; Hazard ratio (95% CI) was calculated from Cox models. ** P*-value<0.001;*** P*-value<0.01;**** P*-value<0.05  Crude model was an unadjusted model;  Model 1 was adjusted with age, gender;  Model 2 was adjusted for age, gender, ethnicity, residence, marital status, education, current smoking, current alcohol drinking, current exercise, BMI, central obesity, ADL disability;  Model 3 was fully adjusted model: adjusted for the confounders in model 2, such as hypertension, diabetes mellitus, heart disease, cerebrovascular disease, respiratory disease, and arthritis(Six diseases were not included in model 4 of Sensitive 2). TG,TC, HDL-C, LDL-C, FBG, SUA, and hs-CRP. | | | | | |


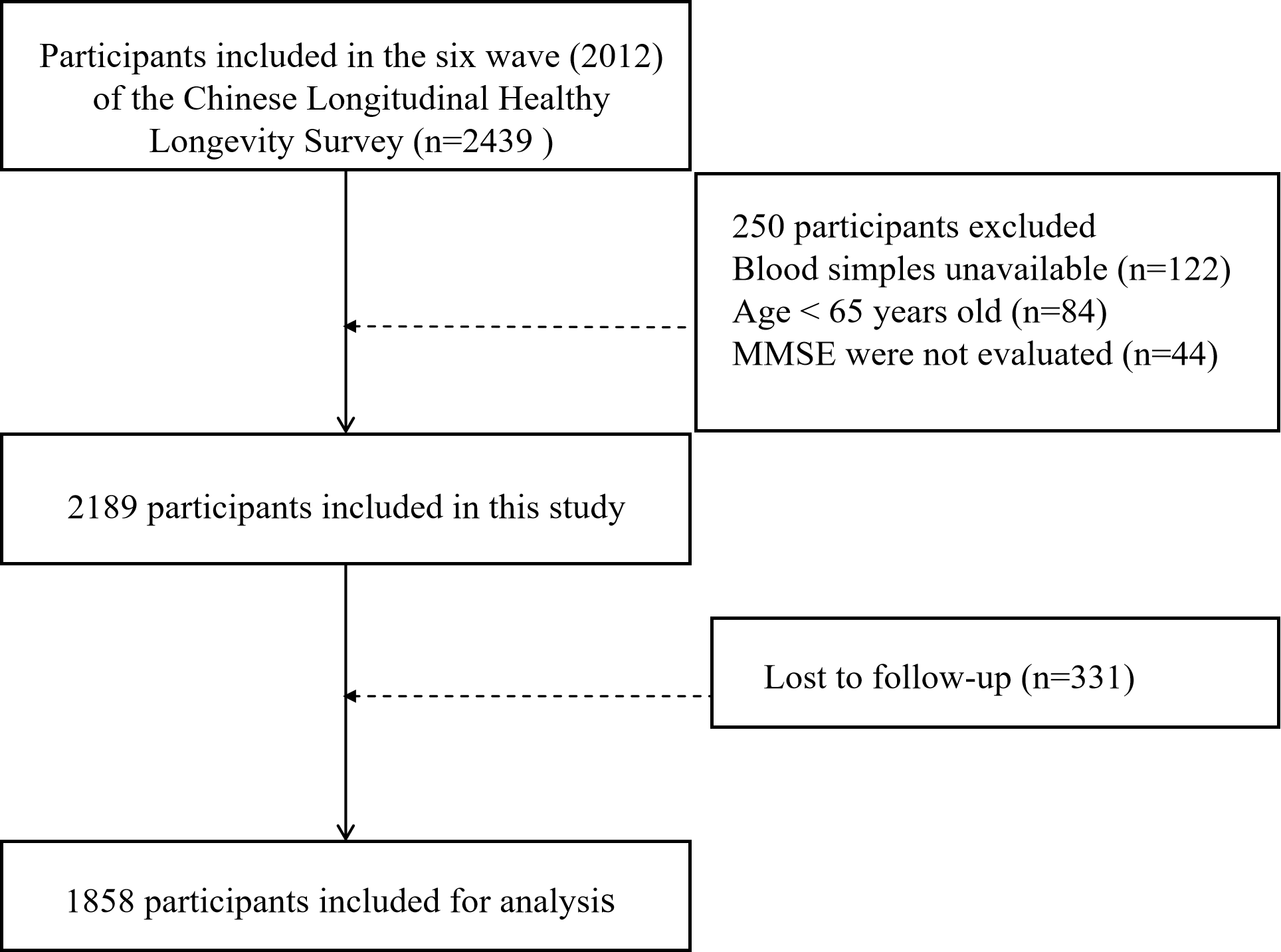
Figure S1.The flow chart of the study. MMSE, Mini-Mental Status Examination
